# A cholinergic trigger drives learning-induced plasticity at hippocampal synapses

**DOI:** 10.1038/ncomms3760

**Published:** 2013-11-12

**Authors:** Dai Mitsushima, Akane Sano, Takuya Takahashi

**Affiliations:** 1 Department of Physiology, Yokohama City University Graduate School of Medicine, 3-9 Fukuura, Yokohama 236-0004, Japan; 2 Department of Physiology and Neuroscience, Kanagawa Dental University, 82 Inaoka-cho, Yokosuka 238-8580, Japan; 3 Department of Systems Neuroscience, Yamaguchi University Graduate School of Medicine, 1-1-1 Minami-kogushi, Ube 755-8505, Japan; 4 Department of Neuroscience, Albert Einstein College of Medicine, 1410 Pelham Pkwy S., Bronx, New York 10461, USA

## Abstract

Learning induces plastic changes in synapses. However, the regulatory molecules that orchestrate learning-induced synaptic changes are largely unknown. Although it is well established that cholinergic inputs from the medial septum modulate learning and memory, evidence for the cholinergic regulation of learning-induced synaptic plasticity is lacking. Here we find that the activation of muscarinic acetylcholine (ACh) receptors (mAChRs) mediates the contextual fear learning-driven strengthening of hippocampal excitatory pyramidal synapses through the synaptic incorporation of AMPA-type glutamate receptors (AMPARs). Contextual fear learning also enhances the strength of inhibitory synapses on hippocampal pyramidal CA1 neurons, in a manner mediated by the activation of, not mAChRs, but, nicotinic AChRs (nAChRs). We observe a significant correlation between the learning-induced increases in excitatory and inhibitory synaptic strength at individual pyramidal neurons. Understanding the mechanisms underlying cholinergic regulation of learning-induced hippocampal synaptic plasticity may help the development of new therapies for cognitive disorders.

## Abstract (web summary)

Cholinergic signalling modulates learning and memory; however, its influence on learning-induced synaptic plasticity is less clear. Mitsushima *et al.* show that acetylcholine simultaneously strengthens both excitatory and inhibitory synapses onto CA1 pyramidal neurons following an inhibitory avoidance task.

## Graphical abstract

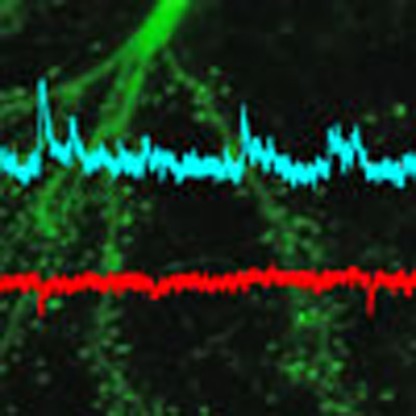

Acetylcholine (ACh) has a crucial role in mediating learning and memory^1^,^2^,^3^,^4^. A number of ACh receptors (AChRs) have been identified and are classified into two large families, muscarinic AChR (mAChRs) and nicotinic AChRs (nAChRs). Although the mAChRs are G-protein-coupled receptors, the nAChRs form ligand-gated ion channels^1^. The cholinergic modulation of synaptic plasticity is well described, including in long-term potentiation (LTP), a cellular model of learning and memory^1^,^5^,^6^,^7^,^8^. However, it is still unclear whether ACh mediates the learning-induced synaptic changes.

Plasticity at excitatory and inhibitory synapses is involved in learning and memory. Experience, such as learning, strengthens excitatory glutamatergic synaptic transmission by driving AMPA (α-amino-3-hydroxy-5-methyl-4-isoxazole propionic acid)-type glutamate receptors (AMPARs) into synapses^9^,^10^,^11^,^12^,^13^,^14^,^15^,^16^,^17^,^18^,^19^. The inhibitory avoidance (IA) task, a contextual fear-learning task, increases AMPARs in CA3-CA1 hippocampal pyramidal synapses, which is required for memory formation^16^. Spatial learning in a water maze increases the frequency but not the amplitude of miniature inhibitory post-synaptic currents (mIPSCs) at hippocampal synapses^20^.

Here we find that mAChR activation mediates the IA learning-induced synaptic delivery of AMPARs to hippocampal CA3-CA1 synapses. IA learning also strengthens inhibitory hippocampal synapses through the activation of nAChRs but not mAChRs. Further, we find significant correlation between the IA-induced increase in miniature excitatory post-synaptic current (mEPSC) and mIPSC amplitudes at individual pyramidal neurons. Thus, ACh balances the excitatory and inhibitory synaptic inputs onto CA1 pyramidal neurons in IA learning through the activation of distinct sets of AChRs.

## Results

### Extracellular ACh level in CA1 increases during learning

**Figure 1:**
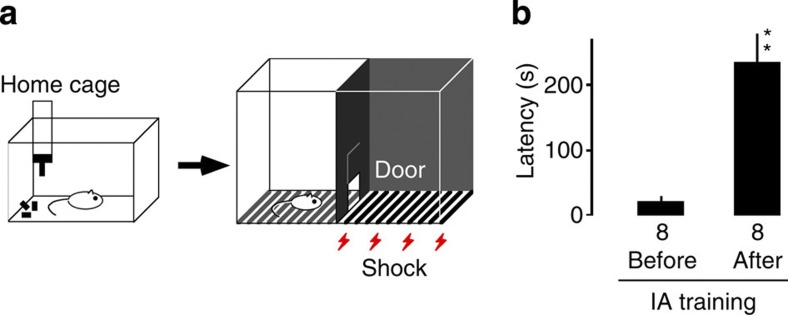
IA task. (**a**) Schema of the light-dark box used for the IA task. Rats were housed in a home cage. On the training day, a brief electrical foot shock (2 s) was applied in the dark box in the shock cage. (**b**) Thirty minutes after IA training, the trained rats consistently showed a longer latency before entering the dark side of the box (**F_1, 14_=26.841, *P*<0.001 versus before, *n*=8, one-way factorial ANOVA, error bars indicate ±s.e.m.).

To investigate learning-induced synaptic modification in the hippocampus, we used the IA task (Fig. 1). In this paradigm, rats are allowed to cross from an illuminated box to a dark box where an electric foot shock is delivered. Thus, rats learn to avoid the dark box and stay in the lighted one, which they would normally not prefer^16^,^17^. The tendency to avoid the dark box therefore indicates the acquisition of contextual memories. The rats avoided entering the dark box when it was associated with a mild electric shock (IA trained), but not those given foot shock without any contextual experience (unpaired) or those allowed to simply explore the experimental cage (walk through) (Fig. 1). Untrained control rats were kept in their home cages and were not exposed to the IA apparatus.

**Figure 2:**
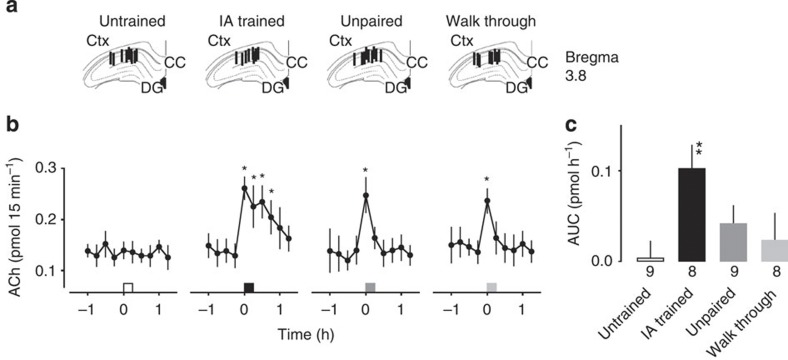
ACh levels in the rat hippocampal CA1 region under different learning conditions. (**a**) Locations of the *in vivo* microdialysis probe in the hippocampal CA1 region. Vertical lines represent the 0.5-mm length of the dialysis membrane. CC, corpus callosum; DG, dentate gyrus. Ctx: cortex. (**b**) Extracellular ACh levels increased significantly during inhibitory avoidance (IA) learning, and remained high for 60 min. In the unpaired or walk-through control animals, the ACh level increased but only transiently. Squares indicate the timing of the behavioural task. (**c**) ACh AUC during and after behavioural tests. The number of rats in each group is shown at the bottom of each bar. *P<0.05 versus pretraining level. One-way ANOVA followed by *post hoc* analysis with the Fisher’s protected least significant difference (PLSD) test. **P=0.0019 versus untrained, 0.048 versus unpaired and 0.018 versus walk through. One-way factorial ANOVA followed by *post hoc* analysis with the Fisher’s PLSD test. Untrained: *n*=9, IA trained: *n*=8, unpaired: *n*=9, walk through: *n*=8, error bars indicate± s.e.m.

As ACh induces LTP in hippocampal slices^21^, we hypothesized that ACh release into the hippocampus triggers the delivery of AMPARs *in vivo*. We therefore examined the extracellular ACh levels in the animals under different learning conditions by *in vivo* microdialysis of the dorsal CA1 (Fig. 2a). Although significant but transient increases in the extracellular ACh levels were observed in the unpaired (*F*_9, 72_=3.830, *P*=0.0005, *n*=9, one-way analysis of variance (ANOVA)) and walk-through animals (*F*_9, 63_=2.137, *P*=0.039, *n*=8, one-way ANOVA), the IA-trained rats displayed a prolonged rise in ACh levels (Fig. 2b, *F*_9, 63_=6.694, *P*<0.0001, *n*=8, one-way ANOVA). No significant change was observed in untrained rats (*F*_9, 72_=0.632, *P*=0.77, *n*=9, one-way ANOVA). To analyse the difference between groups, we calculated the area under the curve (AUC) after the behavioural test (Fig. 2c, *F*_3, 30_=4.130, *P*=0.015, untrained: *n*=9, IA trained: *n*=8, unpaired: *n*=9, walk through: *n*=8, one-way factorial ANOVA). Although the AUC for the IA-trained rats was significantly greater than that for the untrained rats (*P*=0.002 by *post hoc*), the AUC for the unpaired and walk-through rats was not. These results showed that IA learning increases the extracellular ACh levels in the hippocampal CA1 region.

### mAChRs mediate IA-driven excitatory synaptic strengthening

**Figure 3:**
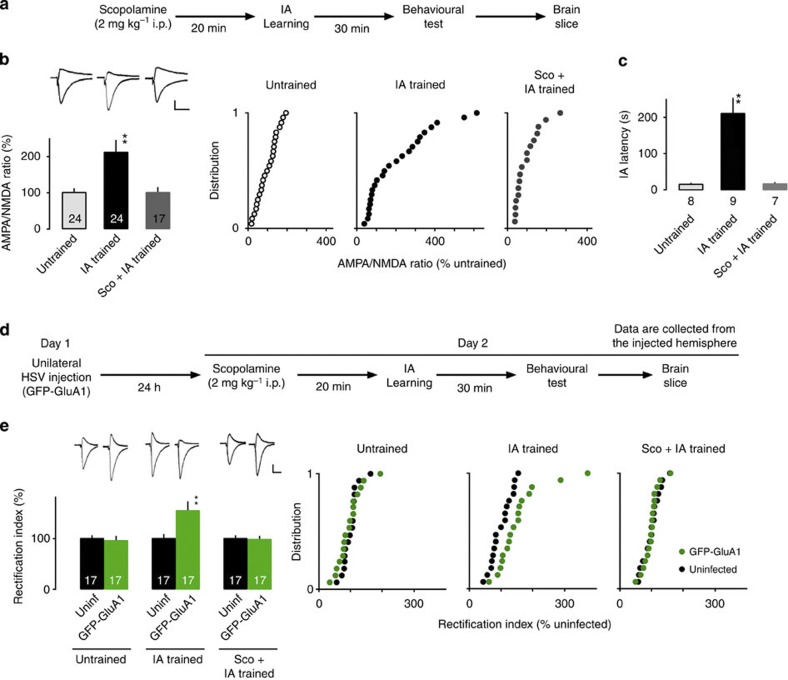
mAChRs mediate the learning-dependent synaptic delivery of AMPARs in CA1 pyramidal neurons. (**a**) Experimental design of the inhibitory avoidance (IA) task. (**b**) AMPA/NMDA ratio in untrained, IA-trained or scopolamine (Sco)-pretreated IA-trained rats (2 mg kg^−1^ i.p.). The AMPA/NMDA ratio in untrained animals was designated as 100%. Sco blocked the IA-training-dependent increase in the AMPA/NMDA ratio (**P=0.0008 versus untrained and 0.0022 versus Sco+IA trained, untrained: *n*=24, IA trained: *n*=24, Sco+IA trained: *n*=17, one-way factorial ANOVA followed by *post hoc* analysis with the Fisher’s PLSD test). Cumulative distributions of the AMPA/NMDA ratio are also shown. (**c**) Latency before re-entering the dark box (IA latency) in untrained, IA-trained and Sco-pretreated IA-trained rats. **P<0.0001 versus untrained (untrained: *n*=8, IA trained: *n*=9, Sco+IA trained: *n*=7, one-way factorial ANOVA followed by *post hoc* analysis with the Fisher’s PLSD test). (**d**) Experimental design of unilateral gene delivery (GFP-GluA1) and the IA task. Data are collected from the injected hemisphere. (**e**) Rectification index (RI: response at –60 mV/response at 40 mV) in untrained, IA-trained or Sco-pretreated IA-trained rats (2 mg kg^−1^ i.p.). The RI of the CA1 pyramidal neurons expressing GFP-GluA1 (green) was normalized to that of nearby uninfected cells (black). **P=0.0003 versus uninfected (*n*=17, paired *t*-test). IA training induced synaptic delivery of the GFP-GluA1, and this delivery was blocked by Sco pretreatment *P*=0.40 (*n*=17, paired *t*-test). Cumulative distributions of the rectification index are also shown. The number of cells (**b**,**e**) or rats (**c**) in each group is shown at the bottom of each bar. Representative traces are shown in top insets. Error bars indicate ±s.e.m. Vertical scale bars, 40 pA; horizontal scale bars, 50 ms.

To examine whether the ACh increase during IA training is required for contextual learning-induced synaptic strengthening, we systemically injected scopolamine (Sco), an mAChR antagonist, 20 min before IA training (Fig. 3a). The IA-trained rats showed an increased AMPAR-mediated synaptic response/NMDA (*N*-methyl-D-aspartate) receptor-mediated synaptic response (AMPA/NMDA) ratio compared with untrained rats, as reported previously^16^ (Fig. 3b, Supplementary Table S1). Pretreatment of Sco fully blocked this IA-induced increase in the AMPA/NMDA ratio (*F*_2, 62_=7.822, *P*=0.0009, untrained: *n*=24, IA trained: *n*=24, Sco+IA trained: *n*=17, one-way factorial ANOVA; Fig. 3b, Supplementary Table S1) and learning (Fig. 3c, *F*_2, 21_=18.133, *P*<0.0001, untrained: *n*=8, IA trained: *n*=9, Sco+IA trained: *n*=7, one-way factorial ANOVA).

As contextual fear conditioning depends on the insertion of GluA1-containing AMPARs into CA1 neurons of the dorsal hippocampus, we further used an electrophysiological tagging technique to detect synaptic insertion of GluA1 receptors^14^,^16^,^22^. For this experiment, GFP-tagged GluA1 (GFP-GluA1) was introduced into the unilateral CA1 region of the dorsal hippocampus by herpes simplex virus (HSV)-mediated *in vivo* gene delivery. Overexpressed recombinant GluA1 forms homomeric receptors and displays little outwards current at positive potentials so that synapses containing recombinant homomeric GluA1 exhibit increased inwards rectification compared with synapses that lack it. The day after the transfection, we subjected the animals to IA training (Fig. 3d). As previously reported^16^, the GFP-GluA1-expressing neurons exhibited increased rectification compared with nearby uninfected neurons in IA-trained (*P*=0.0003, *n*=17, paired *t*-test) but not untrained animals (*P*=0.13, *n*=17, paired *t*-test), showing that IA learning drives GluA1 into hippocampal CA3-CA1 pyramidal synapses (Fig. 3e, Supplementary Table S1). This learning-induced increased rectification of GFP-GluA1-expressing neurons was blocked by the pretreatment of Sco (*P*=0.40, *n*=17, paired *t*-test), suggesting that mAChRs mediate the IA learning-induced synaptic GluA1 delivery (Fig. 3e, Supplementary Table S1).

**Figure 4:**
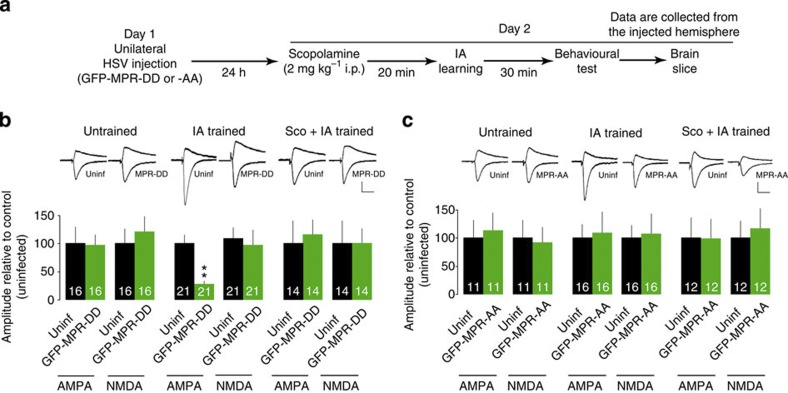
mAChRs mediate learning-dependent synaptic delivery of endogenous GluA1-containing AMPARs. (**a**) Experimental design of unilateral gene delivery (GFP-MPR-DD or -AA) and the inhibitory avoidance (IA) task. Data are collected from the injected hemisphere. (**b**) Unilateral GFP-MPR-DD expression attenuated the learning-dependent synaptic delivery of endogenous GluA1-containing AMPARs in CA1 pyramidal neurons, and scopolamine (Sco) pretreatment with systemic injection blocked this effect. Synaptic transmission from CA3 to CA1 pyramidal neurons was recorded simultaneously from neurons infected with viruses expressing GFP-MPR-DD and nearby uninfected (uninf.) neurons. GFP-MPR-DD expression prevented the potentiation of AMPA transmission in IA-trained rats (**P=0.001 versus uninfected, *n*=21; the Wilcoxon test), but had no effect in untrained (*P*=0.72, *n*=16; the Wilcoxon test) or IA-trained rats in the presence of Sco (*P*=0.30, *n*=14; the Wilcoxon test). NMDA transmission was unchanged by GFP-MPR-DD expression in these groups. (**c**) Unilateral GFP-MPR-AA expression did not affect synaptic transmission in any group. For graphic display, the amplitudes of the corresponding uninfected neurons were designated as 100%. Representative traces are shown in top insets. The number of pairs in each group is shown at the bottom of each bar. Error bars indicate ±s.e.m. Vertical scale bars, 40 pA; horizontal scale bars, 50 ms.

We have previously shown that IA induces synaptic delivery of endogenous GluA1-containing AMPARs at hippocampal CA3-CA1 synapses^16^. To verify whether AChR mediates IA-induced synaptic delivery of endogenous GluA1-containing AMPARs, we expressed mutant form of GluA1. The domain of the GluA1 cytoplasmic tail immediately after the last membrane-spanning region (the membrane-proximal region, MPR: 14 amino acids) is crucial for synaptic GluA1 delivery^16^. Mutating serines in the MPR to phospho-mimicking aspartates (MPR-DD) prevents the synaptic delivery of endogenous GluA1-containing AMPARs, and thus blocks LTP and IA learning, whereas expressing an MPR peptide with the serines mutated to alanine (MPR-AA) has no effect^16^,^23^. Using HSV-mediated *in vivo* gene transfer, we expressed GFP-tagged MPR-DD (GFP-MPR-DD) or MPR-AA (GFP-MPR-AA) in the unilateral dorsal hippocampus, and performed paired whole-cell recordings (Fig. 4a). As previously reported^16^, GFP-MPR-DD- but not GFP-MPR-AA-expressing neurons showed decreased AMPAR-mediated transmission compared with nearby uninfected neurons in IA-trained but not untrained animals (*P*=0.001, *n*=21, the Wilcoxon test), indicating that IA learning drives the endogenous GluA1-containing AMPARs into the CA3-CA1 hippocampal pyramidal synapses (Fig. 4b,c, Supplementary Fig. S1). In the presence of Sco (2 mg kg^−1^ intraperitoneally (i.p.)), we detected no difference in AMPAR-mediated transmission between the GFP-MPR-DD-expressing and nearby uninfected neurons in the IA-trained animals (*P*=0.30, *n*=14, the ilcoxon test), indicating that mAChRs mediate the IA-induced synaptic delivery of endogenous GluA1-containing AMPARs (Fig. 4b and Supplementary Fig. S1a). In contrast, expressing the control fragment (GFP-MPR-AA) did not alter the effect of Sco in IA-trained rats (Fig. 4c and Supplementary Fig. S1b).

**Figure 5:**
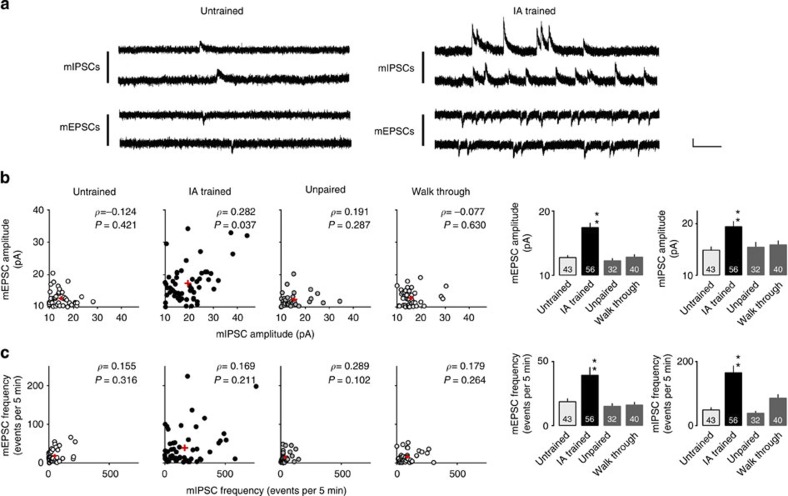
IA learning enhances both excitatory and inhibitory synaptic plasticity. (**a**) Representative traces of miniature EPSCs (mEPSCs) and mIPSCs. mEPSCs at −60 mV and mIPSCs at 0 mV were measured sequentially in the same CA1 pyramidal neuron in the presence of tetrodotoxin (0.5 μM). Vertical scale bar, 20 pA; horizontal scale bar, 200 ms. (**b**) Plots of the mEPSC and mIPSC amplitudes in untrained, trained, unpaired and walk-through rats. Significant correlation between the mEPSC and mIPSC amplitudes in IA-trained rats was observed (*P*=0.037, Spearman’s rank correlation coefficient) **P<0.01 versus untrained (untrained: *n*=43, IA trained: *n*=56, unpaired: *n*=32, walk through: *n*=40, one-way factorial ANOVA followed by *post hoc* analysis with the Fisher’s PLSD test). Contextual learning significantly increased both mEPSC and mIPSC amplitudes. (**c**) Plots of the mEPSC and mIPSC frequency in untrained, trained, unpaired and walk-through rats. Contextual learning significantly increased both mEPSC and mIPSC frequencies. **P<0.01 versus untrained (untrained: *n*=43, IA trained: *n*=56, unpaired: *n*=32, walk through: *n*=40, one-way factorial ANOVA followed by *post hoc* analysis with the Fisher’s PLSD test). Red crosses and bars indicate mean±s.e.m.

**Figure 6:**
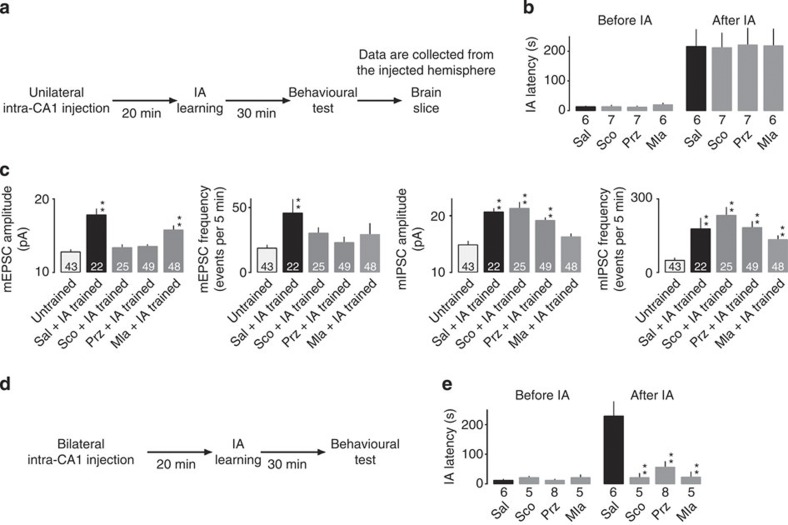
Intra-CA1 injection of cholinergic antagonists blocks learning-enhanced synaptic plasticity. (**a**) Experimental design of unilateral drug infusion into CA1 region and subsequent behavioural test and electrophysiological assay. (**b**) Uilateral intra-CA1 injection of Sco, Prz or Mla did not impair learning. One-way factorial ANOVA. (**c**) Unilateral intra-CA1 injection of Sco or muscarinic M_1_ receptor antagonist (Prz) blocked the learning-dependent increase in the amplitude and frequency of the mEPSC but not of the mIPSC. In contrast, unilateral intra-CA1 injection of a nicotinic α7 receptor antagonist (Mla) blocked the learning-dependent increase in mIPSC amplitude. **P<0.01 versus untrained (untrained: *n*=43, Sal+IA trained: *n*=22 Sco+IA trained: *n*=25, Prz+IA trained: *n*=49, Mla+IA trained: *n*=48, one-way factorial ANOVA followed by *post hoc* analysis with the Fisher’s PLSD test). These data are collected from the injected henisphere. Control experiments without drugs were collected from saline-injected animals. (**d**) Experimental design of bilateral drug infusion into CA1 region and subsequent behavioural test. (**e**) Bilateral intra-CA1 injection of Sco, Prz or Mla impaired learning. **P<0.01 versus saline (Sal: *n*=6, Sco: *n*=5, Prz: *n*=8, Mla: *n*=5, one-way factorial ANOVA followed by *post hoc* analysis with the Fisher’s PLSD test). The number of cells (**c**) or animals (**b**,**e**) is shown at the bottom of the bars. Error bars indicate ±s.e.m.

**Figure 7:**
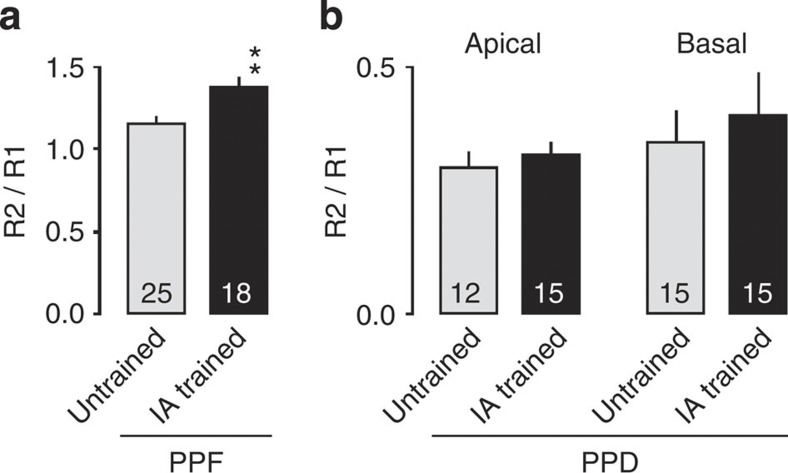
Pre-synaptic alterations in IA learning. (**a**) Paired-pulse facilitation (PPF) at excitatory CA3-CA1 synapses. IA training slightly but significantly increased the rate (R2/R1). **P=0.0024 versus untrained (untrained: *n*=25, IA trained: *n*=18, unpaired two-tailed *t*-test). (**b**) Paired-pulse depression (PPD) at inhibitory synapses in apical or basal dendrites of CA1 neurons. No significant difference was observed. Apical untrained: *n*=12, IA trained: *n*=15, basal untrained: *n*=15, IA trained: *n*=15, unpaired two-tailed *t*-test).

To determine whether the ACh increase during IA conditioning directly regulates synaptic plasticity in the hippocampus, we locally infused Sco or pirenzepine (Prz, an M_1_ mAChR antagonist) into the unilateral hippocampal CA1 region 20 min before IA training. We prepared acute brain slices 30 min after the animals completed the behavioural paradigm, and recorded the mEPSCs at −60 mV in CA1 pyramidal neurons. We detected an overall increase in mEPSC amplitude in the IA-trained animals without drugs compared with the untrained, unpaired and walk-through animals (Fig. 5a,b, *F*_3, 167_=19.834, *P*<0.0001, IA trained: *n*=56, untrained: *n*=43, unpaired: *n*=32, walk through: *n*=40, one-way factorial ANOVA). These data confirmed that the excitatory synapses are strengthened through AMPAR insertion only when the new context is paired with the mild electric shock. We also detected an increase in mEPSC frequency in the IA-trained animals (Fig. 5c, *F*_3, 167_=5.793, *P*=0.0009, IA trained: *n*=56, untrained: *n*=43, unpaired: *n*=32, walk through: *n*=40, one-way factorial ANOVA). As IA learning slightly but significantly increased the paired-pulse facilitation (PPF; *P*=0.0024, untrained: *n*=25, IA trained: *n*=18, unpaired two-tailed *t*-test, Fig. 7, Supplementary Table S2), IA learning may slightly decrease the pre-synaptic glutamate release. Thus, the increased mEPSC frequency after IA learning is probably due to an increased number of functional synaptic contacts in these neurons. Direct unilateral intra-CA1 injection of Sco or Prz infusion blocked IA-induced increase in mEPSC amplitude (*F*_4, 216_=13.144, *P*<0.0001, untrained: *n*=43, Sal+IA trained: *n*=22, Sco+IA trained: *n*=25, Prz+IA trained: *n*=49, one-way factorial ANOVA) and frequency (*F*_4, 216_=2.428, *P*=0.049, untrained: *n*=43, Sal+IA trained: *n*=22, Sco+IA trained: *n*=25, Prz+IA trained: *n*=49, one-way factorial ANOVA) in the injected hemisphere (Fig. 6c) but not in the saline-injected animals (Fig. 6c) or in the non-injected hemisphere of unilaterally injected animals (Supplementary Fig. S2). These unilaterally injected animals successfully learned IA task (Fig. 6b). These results provide further evidence that mAChR (possibly M_1_ mAChR) activation in the hippocampus is required for the learning-dependent strengthening of excitatory CA1 synapses.

To examine whether the local blockade of the mAChRs in the hippocampal CA1 region prevents contextual learning, the same dose of Sco or Prz used in the above electrophysiological experiment was bilaterally infused into the CA1 region before IA conditioning. Although drug treatment did not affect the latency to re-enter the dark box before IA training (*F*_3, 20_=0.750 *P*=0.54, Sal: *n*=6, Sco: *n*=5, Prz: *n*=8, methyllycaconitine (Mla): *n*=5, one-way factorial ANOVA), Sco- or Prz-treated rats exhibited shorter latency than those infused with saline after the IA training (Fig. 6d,e, *F*_3, 20_=7.451, *P*=0.0015, Sal: *n*=6, Sco: *n*=5, Prz: *n*=8, Mla: *n*=5, one-way factorial ANOVA). These data indicate that the activation of mAChRs in the hippocampus is required for the formation of contextual fear memories.

### IA strengthens inhibitory synapses through nAChR activation

As spatial learning in a water maze increases the frequency (but not the amplitude) of mIPSCs (ref. 20), it was possible that plasticity in inhibitory synapses might also be important for creating contextual fear memories. To examine whether IA alters mIPSCs in the same pyramidal neurons in which we recorded the mEPSCs, we changed the holding potential from −60 mV to 0 mV. We detected an increased amplitude (Fig. 5b, *F*_3, 167_=6.397, *P*=0.0004, IA trained: *n*=56, untrained: *n*=43, unpaired: *n*=32, walk through: *n*=40, one-way factorial ANOVA) and frequency (Fig. 5c, *F*_3, 167_=13.786, *P*<0.0001, IA trained: *n*=56, untrained: *n*=43, unpaired: *n*=32, walk through: *n*=40, one-way factorial ANOVA) of mIPSCs in the IA-trained animals compared with the untrained, unpaired and walk-through animals. Some of the CA1 neurons exhibited frequent sequential mIPSCs in the IA-trained rats (Fig. 5a). The paired pulse depression (PPD) was unchanged after IA learning (*P*=0.52 at apical dendrites, *P*=0.63 at basal dendrites, apical untrained: *n*=12, IA trained: *n*=15, basal untrained: *n*=15, IA trained: *n*=15, unpaired two-tailed *t*-test), suggesting that IA learning did not cause significant pre-synaptic alteration (Fig. 7b, Supplementary Table S1). Thus, the increase in mIPSC frequency was probably due to post-synaptic modification.

To examine whether mAChRs mediate the IA-induced strengthening of inhibitory synapses onto CA1 pyramidal neurons, Sco or Prz was unilaterally infused into the CA1 region as described above (Fig. 6a). Although Sal-infused IA-trained rats significantly increased both mIPSC amplitude (*F*_4, 216_=9.418, *P*<0.0001, untrained: *n*=43, Sal+IA trained: *n*=22, Sco+IA trained: *n*=25, Prz+IA trained: *n*=49, one-way factorial ANOVA) and frequency (*F*_4, 216_=8.615, *P*<0.0001, untrained: *n*=43, Sal+IA trained: *n*=22, Sco+IA trained: *n*=25, Prz+IA trained: *n*=49, one-way factorial ANOVA), neither Sco nor Prz unilateral infusion affected the mIPSC amplitude or frequency, suggesting that mAChRs do not mediate the IA-induced enhancement of inhibitory synapses (Fig. 6c).

We next wondered whether nAChRs, the other type of AChRs, mediate the IA-induced alteration of inhibitory synapses. Rats were locally infused in unilateral dorsal hippocampi with the nAChR antagonist Mla and subjected to the IA paradigm. These unilaterally injected animals successfully learned IA task (Fig. 6b). The IA-induced increase in mIPSC amplitude was prevented in the injected hemisphere (Fig. 6c, *F*_4, 216_=9.418, *P*<0.0001, Mla+IA trained: *n*=48, one-way factorial ANOVA) but not in the saline-injected animals (Fig. 6c) or non-injected hemisphere of unilaterally injected animals (Supplementary Fig. S2). These findings together suggest that nAChRs but not mAChRs mediate the IA-induced strengthening of inhibitory synapses onto CA1 pyramidal neurons. Thus, ACh also mediates the IA-induced synaptic modification at inhibitory synapses. Consistent with this finding, bilateral Mla injection into CA1 attenuated IA learning (Fig. 6d,e). Although Mla treatment did not affect the latency to re-enter the dark box before IA training (*F*_3, 20_=0.750 *P*=0.54, Sal: *n*=6, Sco: *n*=5, Prz: *n*=8, Mla: *n*=5, one-way factorial ANOVA), Mla-treated rats showed shorter latency than saline-injected rats after IA training (Fig. 6d,e, *F*_3, 20_=7.451, *P*=0.0015, Sal: *n*=6, Sco: *n*=5, Prz: *n*=8, Mla: *n*=5, one-way factorial ANOVA).

Sequential recordings of mEPSCs (at −60 mV) and mIPSCs (at±0 mV) from the same CA1 neuron revealed diverse responses after IA learning (Fig. 5b). Interestingly, the IA-trained rats showed significant correlation between the mEPSC and mIPSC amplitude, whereas no correlation was detected in the untrained, unpaired or walk-through animals (Fig. 5b). These results suggest there is some learning-dependent interaction between excitatory and inhibitory synaptic strengthening. Considering the effects of cholinergic antagonists (Sco, Prz and Mla in Fig. 6c), ACh may balance the excitatory and inhibitory inputs to hippocampal CA1 neurons in learning through distinct sets of AChRs.

### Long-term maintenance of inhibitory synaptic responses

**Figure 8:**
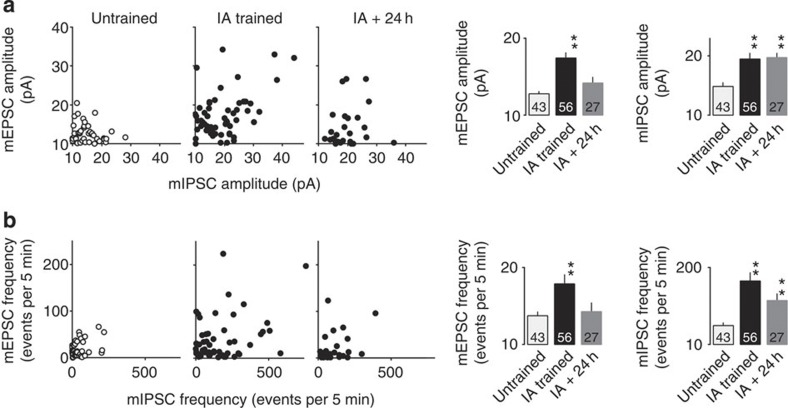
The increase of inhibitory synaptic responses were maintained for more than 24 h after IA learning task. (**a**) Plots of the mEPSC and mIPSC amplitudes in untrained, IA trained and 24 h after the IA-trained rats. Twenty-four hours after learning, significant increase in mIPSC amplitude was still observed (**P<0.0001 versus untrained, untrained: *n*=43, IA trained: *n*=56, IA trained+24 h: *n*=27, one-way factorial ANOVA followed by *post hoc* analysis with the Fisher’s PLSD test), whereas the mEPSC amplitude declined to the basal level (*P*=0.125). (**b**) Plots of the mEPSC and mIPSC frequency in untrained, IA trained and 24 h after the IA-trained rats. Twenty-four hours after learning, significant increase in mIPSC frequency was still observed (**P=0.0003 versus untrained, untrained: *n*=43, IA trained: *n*=56, IA trained+24 h: *n*=27, one-way factorial ANOVA followed by *post hoc* analysis with the Fisher’s PLSD test), whereas the mEPSC frequency declined to the basal level (*P*=0.637). The number of cells in each group is shown at the bottom of each bar. Error bars indicate ±s.e.m.

How was the long-lasting memory stored? We have previously reported that once increased, AMPA/NMDA ratio declines to the basal level 24 h after IA learning^16^. We also measured mEPSC and mIPSC 24 h after IA learning. Whereas the amplitude and the frequency of mEPSC declined to the basal level (presumably because of the homoeostatic mechanism), the amplitude and the frequency of mIPSC remained elevated (Fig. 8). IA learning immediately induces robust excitation as well as inhibition. Elevated excitation is transient, while inhibition remains enhanced. Thus, during initial phase of IA learning, robust excitation is triggered, which might lead to increased inhibition to balance the net activity. Later, only a small number of excitatory synapses could remain strengthened (although average of the amplitude and the frequency of mEPSC declined to the basal level 24 h after IA learning, small number of neurons still exhibited high amplitude and frequency of mEPSC; Fig. 8), whereas majority of strengthened inhibitory synapses were maintained. These synapses might mainly contribute to the encoding of memory.

## Discussion

ACh is a well-known regulator of cognitive functions such as learning and memory. Although many studies have shown ACh’s role in synaptic plasticity *in vitro*, no study has elucidated how ACh regulates learning-driven synaptic modification. In this study, we found that ACh mediates learning-induced strengthening at excitatory and inhibitory synapses through distinct sets of AChRs, revealing novel molecular and cellular mechanisms of learning-dependent synaptic plasticity.

We previously revealed that the synaptic delivery of GluA1-containing AMPARs in the CA1 is required for contextual memory^16^. It is largely accepted that the amount of PPF is inversely related to the initial release probability^24^,^25^. Whereas present results confirm the post-synaptic plasticity at excitatory synapses, learning-dependent increase in the PPF indicates a slight decrease in pre-synaptic glutamate release. Considering that bath application of cholinergic agonist decreases pre-synaptic glutamate release in CA1 neurons^26^, the learning-induced ACh release may contribute to the attenuation of the pre-synaptic glutamate release. Meanwhile, we detected IA-induced increase of the amplitude and frequency of mEPSC at hippocampal CA1 pyramidal neurons. The increase of the amplitude could be because of the additional AMPAR delivery into AMPAR-existing synapses. The increase of the frequency could result from the synaptic AMPAR insertion into silent synapses. Thus, post-synaptic strengthening by synaptic AMPAR delivery could be sufficient to overcome the decrease in pre-synaptic glutamate release. The decrease in glutamate release may participate in the homoeostatic regulation of synaptic strength.

The signalling mechanisms underlying learning-driven ACh-mediated excitatory synaptic modification remain to be determined. The M_1_ mAChR is coupled to a G protein and activates phospholipase C (ref. 1) to generate diacylglycerol, which leads to PKC activation^27^. Activated PKC phosphorylates GluA1 on serine 818, which drives GluA1 into the synapse^23^. In addition, phospholipase C activation produces inositol-1,4,5-triphosphatase and releases Ca^2+^ from intracellular Ca^2+^ stores^28^. The increase in cytoplasmic Ca^2+^ activates CaMKII, which is also crucial for GluA1’s synaptic delivery^29^,^30^,^31^. Both of these pathways could contribute to the learning-driven delivery of GluA1 to the synapse. We also observed that Mla (an α7 nAChR antagonist) application to the unilateral hippocampus blocked the IA-induced increase in mEPSC frequency (and may partially block amplitude with no statistical significance) (Fig. 6c), implying that nAChRs contribute to excitatory synaptic modification. The α7 nAChR is highly permeable to Ca^2+^ (ref. 32), and its activation increases intracellular Ca^2+^ and leads to CaMKII activation. This could also contribute to the ACh-triggered synaptic GluA1 delivery.

At the inhibitory synapses, as the PPD was not altered after IA learning (Fig. 7b), the increased frequency of the mIPSCs owing to IA is probably due to post-synaptic modification. The local unilateral application of an α7 nAChR antagonist to the CA1 region before IA conditioning prevented the learning-dependent increase in mIPSC amplitude (Fig. 6c). Consistent with this, bilateral hippocampal injection of an α7 nAChR antagonist blocked learning itself (Fig. 6d,e), implying that nAChRs can mediate the IA-induced strengthening of inhibitory synapses. In addition, IA-trained rats showed significant correlation between the mEPSC and mIPSC amplitudes (Fig. 5b). Thus, ACh may regulate excitatory/inhibitory balance through distinct sets of AChRs. Meanwhile, we detected a number of neurons that increase excitatory input without enhancing inhibitory input and *vice versa*. Thus, it is also possible that one set of neurons exhibited excitatory changes with inhibitory changes on different set of neurons.

A previous paper showed that the nucleus basalis, the cholinergic forebrain area, is activated during exploring^33^. Further, we found increased levels of ACh in the hippocampus during exploring (Fig. 2). Thus, prolonged elevation of hippocampal ACh levels during IA learning could result from increased activation of the nucleus basalis. In addition to ACh, other neuromodulators such as norepinephrine and serotonin could mediate learning and plasticity^14^,^34^,^35^. It will be crucial to elucidate how brain areas responsible for the regulation of neuromodulators interact and regulate experience-dependent plasticity.

A previous paper reported that a fraction of neurons in the lateral amygdala are involved in cued-fear learning^36^. Consistent with this, there exists heterogeneity of GFP-GluA1-expressing neurons in the CA1 area of hippocampus of IA-trained animals; some of them display siginificant increased rectification, whereas others do not (Fig. 3e right panel). This suggests that a fraction of GFP-GluA1-expressing neurons are involved in IA learning. As both hippocampus and amygdala are involved in IA learning, it will be interesting to examine how neurons involved in IA learning in two different brain areas relate during learning.

In humans, the hippocampal ACh level declines with age, and cholinergic impairment is closely associated with dementia, cognitive impairment and the onset of Alzheimer’s disease. Centrally active acetylcholinesterase inhibitors (donepezil or galanthamine) attenuate such symptoms in mild and moderate-to-severe cases^37^,^38^, indicating the importance of endogenous ACh in human cognitive function. M_1_ mACh activation protects neurons from Aβ-toxicity^39^, and pathogenic Aβ_1−42_ binds to α_7_ nAChRs with high affinity^40^,^41^. Understanding the cholinergic mechanisms underlying memory functions will aid the development of new therapies for cognitive disorders such as Alzheimer’s disease.

## Methods

### Animals

Male Sprague–Dawley rats (postnatal 4–5 weeks of age) were used. The rats were housed under a constant 14-h light/10-h dark cycle (light on: 0500–1900 h) with *ad libitum* access to water and food. All the animal housing and surgical procedures were in accordance with the guidelines of the Institutional Animal Care and Use Committee of the Animal Research Center, Yokohama City University, Kanagawa Dental University, and Yamaguchi University.

### Gene delivery

Recombinant genes (*GFP-GluA1*; *GFP-MPR-DD*; *GFP-MPR-AA*) were cloned by standard methods into an HSV-amplicon vector^18^. The rats were anaesthetized with pentobarbital and positioned in a stereotaxic apparatus (Narishige, Tokyo, Japan). Viral solutions (~4 μl) were pressure injected with a microsyringe through a skull window (~1 mm^2^; Quintessential Stereotaxic Injector, IL, USA). A single unilateral injection into the right hippocampus was performed for whole-cell recordings. The skull and skin were then glued back into position with cyanacrylate glue. Animals infected with the HSV vector were kept in individual cages. Behavioural training was performed 24 h later to permit expression of the recombinant genes.

### Drug injection

Under sodium pentobarbital anaesthesia (30–50 mg kg^−1^, i.p.), a stainless steel guide cannula (outer diameter, 0.51 mm) was implanted stereotaxically into the CA1 region of the dorsal hippocampus. The experiment was performed 1–3 days after the implantation. The coordinates were 3.0 mm posterior to the bregma, 2.0 mm lateral to the midline and 3.8 mm below the surface of the skull. After cannula implantation, a stylet was inserted into the guide until drug injection.

On the day of the experiment, the stylet was replaced with an injector. Approximately 20 min before the IA learning procedure, a general muscarinic receptor antagonist (Sco hydrochloride, Sigma-Aldrich Co., St Louis, MO), muscarinic M_1_ receptor antagonist (Prz dihydrochloride, Sigma-Aldrich Co.) or nicotinic α_7_ receptor antagonist (Mla citrate, Sigma-Aldrich Co.) was directly injected into the CA1 through fine flexible silicone tubing (outer diameter 0.5 mm, Kaneka Medix Co. Osaka, Japan) without restraining the animals (40 μg μl^−1^ per side).

To estimate the spread of microinjected drugs (Sco, molecular weight (MW) 339.8 dalton; Prz, MW 424.32 dalton; Mla, MW 682.8 dalton), same volume (1 μl per side) of cresyl violet (0.1% MW 339.8 dalton) or Luxol fast blue (0.1% MW 736.2 dalton) was directly injected into dorsal CA1 region. Cresyl violet spread ~2,150 μm and Luxol fast blue spread ~2,030 μm of diameter of injected site.

### Electrophysiological recordings

One day after HSV injection, the rats were anaesthetized with ketamine/xylazine or pentobarbital, acute brain slices were prepared and whole-cell recordings were performed as previously described^12^. No viral injection was performed for the analysis of the AMPA/NMDA ratio (Fig. 3b). Briefly, the brain was quickly perfused with ice-cold dissection buffer (25.0 mM NaHCO_3_, 1.25 mM NaH_2_PO_4_, 2.5 mM KCl, 0.5 mM CaCl_2_, 7.0 mM MgCl_2_, 25.0 mM glucose, 110.0 mM choline chloride, 11.6 mM ascorbic acid and 3.1 mM pyruvic acid) and gassed with 5%CO_2_/95%O_2_. Coronal brain slices were cut (350 μm, Leica vibratome, VT-1200S) in dissection buffer and transferred into physiological solution (22–25 °C, 118 mM NaCl, 2.5 mM KCl, 26 mM NaHCO_3_, 1 mM NaH_2_PO_4_, 10 mM glucose, 4 mM MgCl_2_, 4 mM CaCl_2_, pH 7.4, and gassed with 5%CO_2_/95%O_2_). The recording chamber was perfused with physiological solution containing 0.1 mM picrotoxin, 4 μM 2-chloroadenosine at 22–25 °C. For the rectification experiments, we added 0.1 mM D,L-2-amino-5-phosphonopentanoic acid to the perfusate to block NMDA receptors. For the miniature response recordings, we used the physiological solution containing 0.5 μM tetrodotoxin to block Na^+^ channels.

Patch-recording pipettes (4–7 MΩ) were filled with intracellular solution (115 mM caesium methanesulfonate, 20 mM CsCl, 10 mM HEPES, 2.5 mM MgCl_2_, 4 mM Na_2_ATP, 0.4 mM Na_3_GTP, 10 mM sodium phosphocreatine and 0.6 mM EGTA at pH 7.25)^42^. For miniature recordings, we used modified intracellular solution to adjust the reversal potential of the γ-aminobutyric acid-A receptor response (127.5 mM caesium methanesulfonate, 7.5 mM CsCl, 10 mM HEPES, 2.5 mM MgCl_2_, 4 mM Na_2_ATP, 0.4 mM Na_3_GTP, 10 mM sodium phosphocreatine, 0.6 mM EGTA, pH 7.25). Whole-cell recordings were obtained from infected or uninfected CA1 pyramidal neurons of rat hippocampus with an Axopatch–700B amplifier (Axon Instruments). There were no significant differences in input or series resistance among groups. Bipolar tungsten stimulating electrodes were placed in CA1 ~200–300 μm lateral from the recorded cells. The stimulus intensity was increased until a synaptic response of amplitude >~10 pA was recorded. When recording simultaneously from two cells, the stimulus intensity was increased until both cells showed a response >~10 pA. Synaptic AMPA receptor-mediated responses at –60 and +40 mV were averaged over 50–100 trials, and their ratio (averaged response at −60 mV/+40 mV) was used as an index of rectification. For paired recordings, infected and nearby uninfected cells (~100 μm) were accessed as whole cells, and the synaptic response to a stimulus was recorded from both cells simultaneously.

The AMPA/NMDA ratio was calculated as the ratio of the peak current at −60 mV to the current at +40 mV 150 ms after stimulus onset (40–60 traces averaged for each holding potential). For the miniature recordings, the mEPSC (−60 mV holding potential) and mIPSC (0 mV holding potential) were recorded for 5 min in the same CA1 neuron. Bath application of an AMPA receptor blocker (CNQX, 10 μM) or γ-aminobutyric acid-A receptor blocker (bicuculline methiodide, 10 μM) completely blocked the mEPSC (at −60 mV) or mIPSC (at 0 mV) events, respectively. To evaluate the paired-pulse ratio from the EPSC or IPSC average, 30–60 sweeps were recorded with paired stimuli at 100-ms intervals. The EPSC or IPSC amplitudes were measured from the peak of the post-synaptic current to the basal current level immediately before the electrical stimulation.

### *In vivo* microdialysis

Under sodium pentobarbital anaesthesia (30–50 mg kg^−1^, i.p.), a stainless steel guide cannula (outer diameter, 0.51 mm) was implanted stereotaxically into the right side of the dorsal hippocampus. After cannula implantation, a stylet was inserted into the guide until the microdialysis was performed. Although the rats were reared and housed socially, after surgery each rat was individually housed in a cylindrical plastic cage (diameter=35 cm, height=45 cm).

The experiment was performed in an electromagnetic- and sound-shielded room (length 1.2 m, width 2.2 m and height 2.3 m)^43^. The stylet was replaced with a microdialysis probe the day before the experiment (outer diameter=0.31 mm, AI-4-0.5, Eicom Co., Kyoto, Japan). A two-channel fluid swivel device (SSU-20; Eicom Co.) was connected to the inlet and outlet of the probe. During the experiment, an artificial cerebrospinal fluid solution (147 mM NaCl, 4 mM KCl, 1.2 mM CaCl_2_ and 0.9 mM MgCl_2_) was infused through the dialysis probe with a 0.5-mm-long semipermeable membrane at a rate of 1.2 μl min^−1^ using a microdialysis pump (CMA/102, Carnegie Medicin, Stockholm, Sweden). The rats were housed individually in a cage, and the dialysis was performed under unanesthetized, freely moving conditions. After an overnight stabilization period, the dialysates were automatically collected into an autoinjector (24 μl; EAS-20, Eicom Co.) every 15 min, and the same volume of ethylhomocholine solution (100 nM) was mixed in as the internal standard. This mixture was injected directly onto an HPLC column every 15 min^44^.

### IA learning

On the training day, the rats were moved into an electromagnetic- and sound-shielded room (length 1.2 m, width 2.2 m and height 2.3 m) with an IA training apparatus (length 25 cm, width 62 cm and height 45 cm). The apparatus is a two-chambered Perspex box consisting of a lighted safe side and a dark shock side, separated by a trap door. During training, the IA-trained rats were placed in the safe side of the box facing a corner opposite the door. After the trap door was opened, rats could enter the dark box at will. The latency before entering the novel dark box was measured as a behavioural parameter (latency before IA learning). Four seconds after the animals entered the dark side, we closed the door and applied a scrambled electrical foot shock (2 s, 1.6 mA) via electrified steel rods in the floor of the box. The rats were kept in the dark compartment for 10 s before being returned to their home cage.

The training procedure for the control groups was as follows: the unpaired control rats (foot shock only) were placed in the box in the dark side and subjected to a scrambled electrical foot shock (2 s, 1.6 mA) without any contextual experience. The walk-through control rats were placed in the IA training apparatus and allowed to explore for 1 min, without shock. The untrained control rats were not removed from their home cages.

Thirty minutes after the procedure described above, the rats were placed in the lighted side. The latency before entering the dark box was measured as an indicator of learning performance (latency after IA learning). The rats were then killed with an overdose of pentobarbital. For the untrained control, rats were injected with the same dose of anaesthesia in their home cage.

### Biochemical analysis of ACh

ACh was quantified by combining HPLC, an enzyme reaction, and electrochemical detection (HTEC-500, Eicom Co.). A solution of 0.1 mM Na_2_HPO_4_ (pH 8.5) and 200 mg l^−1^ sodium 1-decanesulfonate (Aldrich Chemical Company, Inc., Milwaukee, WI) was delivered as the HPLC mobile phase at 150 μl min^−1^. After sample separation in a styrene polymer column (AC-GEL, Eicom Co.), the ACh was converted to hydrogen peroxide by a post-column enzyme reactor (AC-ENZYMPAK, Eicom Co.) containing immobilized acetylcholinesterase and choline oxidase. The hydrogen peroxide was detected with an electrochemical detector, with a minimum detectable amount of 5–10 fmol per sample.

To calculate the recovery rate of each dialysis probe, standard samples were also infused through the probe *in vitro*. The amount of ACh collected every 15 min was divided by the *in vitro* recovery rate to estimate the extracellular ACh level.

### Statistics

Extracellular ACh levels were analysed by one-way ANOVA with repeated measures followed by *post hoc* analysis with the Fisher-protected least significant difference test, where the variable was time. To evaluate the difference between groups, we calculated the baseline levels of ACh before the behavioural test, and the response was calculated as the AUC for each rat. The AUC, AMPA/NMDA ratio, IA latency, mEPSC and mIPSC data were analysed by one-way factorial ANOVA where the variable was the treatment group. The ANOVA was followed by *post hoc* analysis with the Fisher’s protected least significant difference test. The rectification index was analysed by a paired *t*-test. Cumulative distribution was analysed by the Kolmogorov–Smirnov test. The paired recording data were analysed by the Wilcoxon non-parametric test. The ratio of PPF or PPD (R2/R1) was analysed by unpaired two-tailed *t*-test. To evaluate the correlation between mEPSC and mIPSC parameters, Spearman’s rank correlation coefficient was calculated. *P*<0.05 was considered statistically significant.

## Author contributions

D.M. and A.S. performed the experiment. T.T. and D.M. designed the experiment and wrote the manuscript. T.T and D.M. co-managed the project.

## Additional information

**How to cite this article:** Mitsushima, D. *et al.* A cholinergic trigger drives learning-induced plasticity at hippocampal synapses. *Nat. Commun.* 4:2760 doi: 10.1038/ncomms3760 (2013).

## Supplementary Material

Supplementary Information Supplementary Figures S1-S2 and Supplementary Tables S1-S2
