# Supplementary material for: A cholinergic trigger drives learning-induced plasticity at hippocampal synapses
Source: Nat Commun. 2013 Nov 12;4:2760. doi: 10.1038/ncomms3760 (PMC3831287; doi:10.1038/ncomms3760)
Supplement: Supplementary Information — Supplementary Figures S1-S2 and Supplementary Tables S1-S2 [file ncomms3760-s1.pdf]

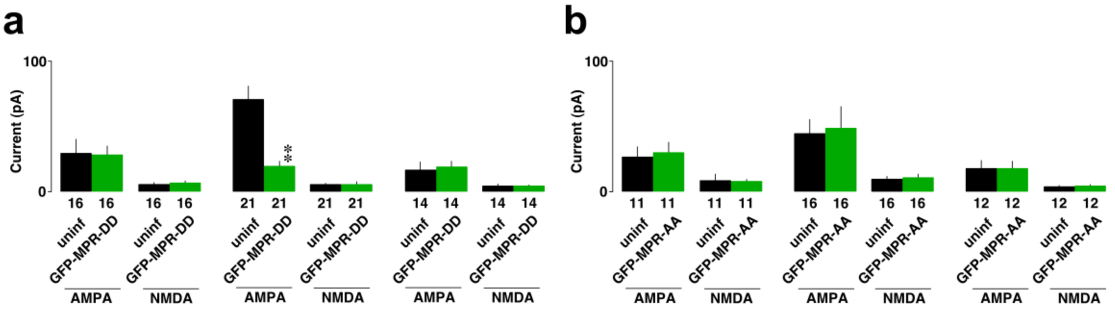

Supplementary Figure S1 Absolute values of synaptic currents in Figure 4b and 4c

(a) GFP-MPR-DD expression attenuated the learning-dependent synaptic delivery of endogenous GluA1-containing AMPARs in CA1 pyramidal neurons, and scopolamine (Sco) pretreatment blocked this effect. Synaptic transmission from CA3 to CA1 pyramidal neurons was recorded simultaneously from neurons infected with viruses expressing GFP-MPR-DD and nearby uninfected (uninf.) neurons. GFP-MPR-DD expression prevented the potentiation of AMPA transmission in IA-trained rats (** P = 0.001 *vs* uninfected, n=21, Wilcoxon test), but had no effect in untrained or IA-trained rats in the presence of Sco. NMDA transmission was unchanged by GFP-MPR-DD expression in these groups. (b) GFP-MPR-AA expression did not affect synaptic transmission in any group. The number of cells is shown at the bottom of the bars. Error bars indicate ± SEM.

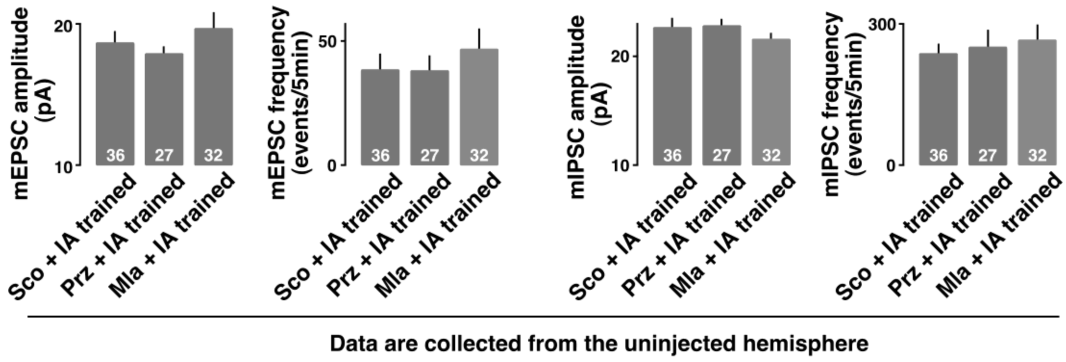

Supplementary Figure S2 Unilateral intra-CA1 injection of cholinergic antagonists did not affect the learning-enhanced synaptic plasticity in contralateral CA1 neurons

Data were collected from CA1 neurons in uninjected hemisphere. Neither Sco, Prz nor Mla injection affects the learning-dependent increase in mEPSC or mIPSC responses (one-way factorial ANOVA). The number of cells is shown at the bottom of the bars. Error bars indicate ± SEM.

Supplementary Table S1. Absolute values of synaptic currents

		Mean currents of Figure 3b (pA)	
		AMPA (Schaffer)	NMDA (Schaffer)
untrained		41.2 ± 5.3 (24)	9.3 ± 1.9 (24)
IA trained		97.5 ± 18.1 (24)	9.1 ± 1.2 (24)
Sco + IA trained		56.8 ± 10.0 (17)	9.9 ± 1.8 (17)

		Mean currents of Figure 3e (pA)	
	holdings (mV)	uninfected	GFP-GluA1
untrained	-60	93.8 ± 18.2 (17)	101.4 ± 16.2 (17)
untrained	40	23.2 ± 5.0 (17)	43.6 ± 6.5 (17)
IA trained	-60	151.0 ± 25.9 (17)	229.8 ± 48.9 (17)
IA trained	40	68.1 ± 20.5 (17)	110.8 ± 26.4 (17)
Sco + IA trained	-60	106.2 ± 16.1 (17)	93.2 ± 9.8 (17)
Sco + IA trained	40	23.7 ± 5.2 (17)	19.9 ± 4.3 (17)

		Mean currents of Figure 7b (pA)	
		$GABA_A$ (apical)	$GABA_A$ (basal)
untrained		20.7 ± 6.1 (12)	22.7 ± 9.1 (15)
IA trained		26.4 ± 8.8 (15)	13.9 ± 5.3 (15)

Number of cells were shown in parentheses.

Supplementary Table S2. Absolute values of paired-pulse facilitation

	Mean currents of Figure 7a (pA)	
	R1	R2
untrained	29.5 ± 2.5 (25)	33.8 ± 3.0 (25)
IA trained	26.2 ± 4.2 (18)	34.3 ± 4.6 (18)

Number of cells were shown in parentheses.
